# Recovery planning towards doubling wild tiger *Panthera tigris* numbers: Detailing 18 recovery sites from across the range

**DOI:** 10.1371/journal.pone.0207114

**Published:** 2018-11-08

**Authors:** Abishek Harihar, Pranav Chanchani, Jimmy Borah, Rachel Jane Crouthers, Yury Darman, Thomas N. E. Gray, Shariff Mohamad, Benjamin Miles Rawson, Mark Darmaraj Rayan, Jennifer Lucy Roberts, Robert Steinmetz, Sunarto Sunarto, Febri Anggriawan Widodo, Meraj Anwar, Shiv Raj Bhatta, Jayam Peter Prem Chakravarthi, Youde Chang, Gordon Congdon, Chittaranjan Dave, Soumen Dey, Boominathan Durairaj, Pavel Fomenko, Harish Guleria, Mudit Gupta, Ghana Gurung, Bopanna Ittira, Jyotirmay Jena, Alexey Kostyria, Krishna Kumar, Vijay Kumar, Phurba Lhendup, Peiqi Liu, Sabita Malla, Kamlesh Maurya, Vijay Moktan, Nguyen Dao Ngoc Van, Karmila Parakkasi, Rungnapa Phoonjampa, Worrapan Phumanee, Anil Kumar Singh, Carrie Stengel, Samundra Ambuhang Subba, Kanchan Thapa, Tiju C. Thomas, Christopher Wong, Michael Baltzer, Dipankar Ghose, Sejal Worah, Joseph Vattakaven

**Affiliations:** 1 WWF-Tigers Alive Initiative, New Delhi, India; 2 WWF-India, New Delhi, India; 3 WWF-India, Assam, India; 4 WWF-Greater Mekong Program, Phnom Penh, Cambodia; 5 WWF-Russia, Amur branch, Vladivostok, Russia; 6 WWF-Malaysia, Kuala Lumpur, Selangor, Malaysia; 7 WWF-Vietnam, Hanoi, Vietnam; 8 Durrell Institute of Conservation and Ecology, School of Anthropology and Conservation, University of Kent, Canterbury, United Kingdom; 9 WWF-Tigers Alive Initiative, Phnom Penh, Cambodia; 10 WWF-Thailand, Bangkok, Thailand; 11 WWF-Indonesia, Jakarta, Indonesia; 12 WWF-Indonesia, Central Sumatra Program, Pekanbaru, Riau, Indonesia; 13 WWF-India, Terai Arc Landscape Office, Haldwani, Uttarakhand, India; 14 WWF-Nepal, Programme Office, Kathmandu, Nepal; 15 WWF-India, Western Ghats Nilgiris Landscape Office, Coimbatore, Tamil Nadu, India; 16 WWF-China, Changchun, Jilin Province, P. R. China; 17 WWF-India, Satpura Maikal Landscape Office, Mandla, Madhya Pradesh, India; 18 WWF-India, Satpura Maikal Landscape Office, Jabalpur, Madhya Pradesh, India; 19 WWF-India Terai Arc Landscape Office, Pilibhit, Uttar Pradesh, India; 20 WWF-India, Programme Office, Dehradun, Uttarakhand, India; 21 WWF-India, Satpura Maikal Landscape Office, Balaghat, Madhya Pradesh, India; 22 WWF-India, Western Ghats Nilgiris Landscape Office, Bhavanisagar, Tamil Nadu, India; 23 WWF-Bhutan, Program Office, Thimphu, Bhutan; 24 WWF-Tigers Alive Initiative, Jakarta, Indonesia; 25 WWF-Tigers Alive Initiative, Washington-D.C., United States of America; 26 WWF-Tigers Alive Initiative, Singapore; Michigan Technological University, UNITED STATES

## Abstract

With less than 3200 wild tigers in 2010, the heads of 13 tiger-range countries committed to doubling the global population of wild tigers by 2022. This goal represents the highest level of ambition and commitment required to turn the tide for tigers in the wild. Yet, ensuring efficient and targeted implementation of conservation actions alongside systematic monitoring of progress towards this goal requires that we set site-specific recovery targets and timelines that are ecologically realistic. In this study, we assess the recovery potential of 18 sites identified under WWF’s Tigers Alive Initiative. We delineated recovery systems comprising a source, recovery site, and support region, which need to be managed synergistically to meet these targets. By using the best available data on tiger and prey numbers, and adapting existing species recovery frameworks, we show that these sites, which currently support 165 (118–277) tigers, have the potential to harbour 585 (454–739) individuals. This would constitute a 15% increase in the global population and represent over a three-fold increase within these specific sites, on an average. However, it may not be realistic to achieve this target by 2022, since tiger recovery in 15 of these 18 sites is contingent on the initial recovery of prey populations, which is a slow process. We conclude that while sustained conservation efforts can yield significant recoveries, it is critical that we commit our resources to achieving the biologically realistic targets for these sites even if the timelines are extended.

## Introduction

Less than 3200 wild tigers (*Panthera tigris*) were estimated to occupy merely ~5% of their historic range in 2010 [1–4]. In response, the 13 tiger range countries (TRCs) with support from international donor and conservation agencies committed to a 12-year goal of doubling wild tiger numbers by 2022 [1]. Based on an evaluation of potential tiger numbers (to at least 6000 individuals by TRCs and conservation agencies) that could be supported across the range, this goal, represents the highest level of ambition and commitment required to turn the tide for tigers in the wild [1]. To achieve this, the Global Tiger Recovery Program (GTRP), which has been central to concentrating efforts and mobilizing support, outlined an action plan to strengthen national policies, build institutional frameworks and secure financial commitments. However, to ensure efficient and targeted implementation of conservation actions alongside systematic monitoring of progress [5], it is critical to set site-specific goals based on ecologically realistic targets and timelines estimated from site-level information. Therefore, in this study we set site-level targets and assess potential timelines of population recovery for tigers across 18 recovery sites identified under the World Wide Fund for Nature’s (WWF) Tigers Alive Initiative (a range-wide long-term endeavour that aims to promote tiger recovery towards achieving this global target; S1 Text). We do so by defining recovery systems using a standardized set of characteristics, evaluating baseline and target population sizes of tigers, and assessing how this conservation program can contribute not just to the goal of doubling tiger numbers, but also ensure sustained recovery and persistence of these populations.

Recovery entails increasing population abundance, ensuring viability, and eliminating or minimizing threats to improve the likelihood of the species’ persistence in their landscapes [5–7]. Key factors that have facilitated persistence and recovery of tiger populations are the presence of inviolate habitats that provide secure breeding refuges, adequate prey availability, effective protection from poaching, low conflict with people, functional connectivity to a source population to facilitate colonization and local community participation in conservation [8–11]. Population recovery in isolated sites, where tigers are locally or functionally extinct, have additionally depended both on responsive governance that has ensured that the causes of extinction have been addressed, and on timely re-introduction/supplementation (e.g. Panna and Sariska Tiger Reserves, India [12–14]).

### Population recovery planning for tigers

Prior to initiating recovery measures, it is critical to demarcate the spatial extent within which interventions should be carried out, establish baselines, set realistic population targets and assess potential timelines towards achieving the target [7]. The 18 recovery sites (identified by us), embedded within priority Tiger Conservation Landscapes (TCLs; [2]) where conservation intervention is essential to enable tiger recovery, currently sustain fewer tigers than their carrying capacity, receive less management attention than proximate source sites and hold the potential to deliver significant return on conservation investments (S1 Table). Recognizing that both intensive site-level protection and landscape-scale interventions to maintain permeable corridors for tiger movement are essential [15,16], we delineated recovery systems typically consisting of a recovery site, source site and support region (see Table 1 for definitions) based on site-specific ecological factors. We established baselines of tiger and prey abundance within the recovery systems, by compiling the most current estimates. We then identified realistic recovery targets for the sites under varying scenarios of data availability. Where robust site-level baselines were available (tiger and prey densities as well as covariates, which influence these including habitat features and threats), we assessed the potential timelines for recovery.

**Table 1:** Component parts of a recovery system.

Source site (Definition from Walston *et al*.[15])	A site with higher densities of tigers than in the larger tiger-permeable landscape within which it is embedded with evidence of breeding and the potential to maintain a demographically viable cluster of >25 breeding females, alone or combined with other connected source sites in the same landscape. The site benefits from a legal framework (existing or under development) for the prevention of poaching or hunting of tigers and their prey, with existing/proposed wildlife protection capacity and genuine government/social commitment to preventing further human in-migration and/or infrastructure development.
Recovery site (Definition adapted from Ranganathan *et al*.[17] and Seidensticker *et al*.[18])	A site with lower densities of tigers than in the larger tiger-permeable landscape within which it is embedded that has the potential to significantly contribute (i.e. augment the source) towards maintaining a demographically viable population and where threats that depress population densities still persist. The management attention in terms of financial commitment and/or protection capacity is lesser in comparison to the adjoining source or high density populations in the same landscape.
Support region	Areas that either provide crucial connectivity between the source and the recovery site (such as movement corridors, other critical habitat units) and/or buffer the recovery site. These regions could comprise several management units (e.g. protected areas, forest reserves and concessions), but require targeted management interventions to ensure the viability of corridors or additional habitat to accommodate spill-over populations.

## Materials and methods

We identified 18 recovery sites that currently sustain fewer tigers than they can potentially support and receive less management attention (in terms of financial commitment and/or protection capacity) than the source sites within these landscapes (S1 Table). However, it is critical to note that these 18 sites are a subset of a larger number of potential recovery sites, which Tigers Alive Initiative may include in the future. For this assessment we, (1) demarcated the spatial extents of recovery systems based on landscape configuration and known patterns of tiger and/or prey occurrence and abundance; (2) determined baseline estimates of tiger and prey abundance within the recovery systems; and (3) identified realistic recovery targets for the sites under varying scenarios of data availability.

### (1) Delineating recovery systems

In general, the recovery system comprised of a source site, recovery site and support region (see Table 1 for definitions). First we compiled spatial information from TCLs and identified known source sites and recovery sites [15,17,18]. Based on known patterns of tiger and/or prey occurrence and abundance, identified recovery sites fell within five broad groups (S2 Table):

- Source sites (n = 6)—These are sites (or sections of sites) identified by Walston *et al*. [15] as a "source", which can host significant recoveries themselves.
- Sites abutting functional source sites (n = 5)—These are recovery sites that are abutting, or more specifically sharing a boundary with, previously identified source sites [15] that are known to support viable tiger populations.
- Disjunct source and recovery sites (n = 2)—These are recovery sites that are embedded within TCLs with functional sources sites, but are separated from such source sites by human land use areas.
- Standalone recovery sites in extant range (n = 3)—these are recovery sites that are embedded within TCLs, where connectivity to functional source sites is weak/too distant, and
- Recovery sites in landscapes where tigers are locally/functionally extinct (n = 2)—these are recovery sites that are embedded within TCLs with no current evidence of breeding populations.

Support regions were delineated with the purpose of either serving as a buffer around the recovery site or providing connectivity between the source and the recovery site. In circumstances where the recovery sites were in close proximity to known sources, adjacent forests were delineated as the support region (n = 11; S2 Table). And, in cases where the source and recovery site were disjoint (n = 2), a circuit theory based connectivity analysis implemented in CIRCUITSCAPE v.4 was conducted to identify the support region [19,20].

The two sites (Balaghat and Achanakmar), where connectivity modelling was employed, are a part of the Kanha-Phen Global Priority TCL in the central Indian region. Several studies aimed at understanding the patterns of connectivity for tigers residing in protected areas of the region have been conducted in the recent past [21–28]. These studies indicate varying degrees of structural and genetic connectivity between populations and highlight the importance of corridors in ensuring viability and persistence of the species in the landscape. However, for the purpose of this study, we reassessed structural connectivity as (a) studies have so far considered Balaghat as a part of connecting forests and not a potential population, (b) Achanakmar, with a small population has often not been included in connectivity analysis (with the exception of [24,25]) and (c) our aim was to identify land management units to include as part of the support regions for the recovery sites.

In order to characterize the resistance of the landscape we used the Human Footprint index (HF)—a global dataset, available at a spatial resolution of 1-km grid cells, created with data layers covering built environments, croplands, pasture lands, human population density, nightlights, railways, major highways and navigable waterways [29,30]. We used this data layer as it combines multiple proxies of human influence that have been shown to affect tiger movement in the region [22–28] as well as terrestrial mammalian movement globally [31]. The HF index varies from 0 (natural environments) to 50 (high-density built environments), for the analysis we rescaled resistance values to a scale of 0–100. Given that both recovery systems were within the same TCL and shared a source sites (i.e. Kanha), we conducted a single analysis where we considered Balaghat, Achanakmar, Kanha, and Pench as ‘nodes’. The boundary shapefiles of these sites were rasterized to a spatial resolution of 1-km grid cells and HF resistance layer was clipped to an 87,000km^2^ extent defining our area of interest. A pairwise algorithm was chosen to generate estimates of current flow density. As we recognize that tigers disperse across/use a wide variety of land categories [26,32–40], we did not mask our models using existing forest cover. Although agricultural/human land use matrix does not typically represent tiger habitat, we describe it as the support region for tigers in disjunct habitat patches because the persistence of metapopulations is contingent on landscape permeability of the matrix. Following the analysis, we overlaid a spatial layer of land management units in the region to objectively identify all land areas (state and private landholdings) permeable to movement of tigers.

### (2) Assessing the status of tigers and their prey

Based on the most recent site-specific data available to us, we compiled estimates of tiger (adult individuals/100km^2^) and wild prey (individuals/km^2^) densities as a metric to evaluate their status at the source site, recovery site and support region (S3 Table).

Across the tigers’ range it is widely accepted that tiger density can reliably be estimated using photographic capture-recapture based sampling methodologies [41–43]. Nevertheless, estimates of tiger density are known to vary on account of the analytical technique used (either the mean maximum distance moved estimators; MMDM or ½MMDM methods or spatially explicit capture-recapture procedures; SECR models) and it has been demonstrated that SECR models outperform MMDM estimators by providing estimates closest to true densities [44–47]. Therefore, we primarily collated estimates derived from an SECR analysis of photographic capture-recapture data (using either a Frequentist or Bayesian inference method) (S3 Table).

To assess the status of wild prey species, several studies employ distance based line transect methodologies that estimate population density [48,49]. However, these methods require considerable number of independent detections to successfully model the probability of detection and hence are not suited in areas of either low density or low visibility (e.g. sites in rainforests/tropical evergreen forests, temperate coniferous and steppe habitats). In such sites, species occupancy or indirect track counts (i.e. the proportion of area occupied [50] or track based estimates of density [51,52] estimated while accounting for imperfect detectability (e.g. of signs) were considered (S3 Table).

### (3) Setting recovery targets using the best available data

Prior studies set recovery targets by assigning potential tiger densities determined for major habitat types/biomes (e.g. [16]). These estimates often characterize the maximum density attained within well-protected regions of these biomes and are hence not representative of the heterogeneity in habitat features, anthropogenic influences and management regimes typically present across landscapes. Ideally realistic recovery targets are set by gaining a comprehensive understanding of site-specific baselines of tiger and prey densities as well as covariates which influence these including habitat features and threats (e.g. [53]). However, such data are rarely available across the tiger’s range and hence we had to set population recovery targets for each of the identified recovery sites using one of several approaches.

We adopted two broad strategies to set realistic recovery targets, i.e. the number of individuals that a site can support provided effective implementation of conservation actions. These strategies were based on the resolution at which data on tiger and prey abundances, and associated habitat and threat covariates were available: (a) site-level (n = 4), and (b) landscape-level (n = 14) (S4 Table).

#### Setting recovery targets with site-level data and estimating timelines for recovery

For four recovery sites, we had reliable estimates of initial population size (*N*_0_) and site-specific data on prey availability to arrive at realistic population targets (*K*; carrying capacity). Using this information, we performed Population Viability Analysis (PVA) in program VORTEX v10 [54–56], to understand relative trade-offs between alternative management actions aimed at recovering tigers at the site. Demographic parameters used in the PVA were derived from multiple sources [57–62]. Inbreeding is often considered important in conservation biology, especially with the case of re-introduction /supplementation programs. However, in the absence of species-specific data we used the value of 6.26 for lethal equivalents, which is the combined mean effect of inbreeding on fecundity and first year survival reported by O’Grady et al., [63]. Lack of suitable data also prevented us from modelling density dependence at population sizes below *K*. We also did not include periodic environmental catastrophe, although they contribute significantly to the population growth or decline, where operational. An example of such an event affecting large carnivores is the Canine Distemper Virus epidemic that almost wiped out the Serengeti lion population in 1994, a threat that has been identified in tiger populations [64–66]. For the analysis, all simulations were run for 500 iterations for a period of 100 years and extinction was defined as the condition when the population is reduced to only one sex. A unique set of scenarios was evaluated for each of the sites based on relevant prior information. It was only for these four sites (Srepok, Cambodia; Shuklaphanta, Nepal; Nandhaur & Western Rajaji; India) that we were able to assess the potential timelines for recovery. Finally, a sensitivity analysis was performed for all uncertain parameters pertaining to reproductive and mortality rates. Detailed description of the VORTEX model and unique set of scenarios are provided in S2 Text.

#### Setting recovery targets with landscape-level data

For 14 recovery sites, we used data obtained from similar habitat types, source sites, other protected areas or published habitat suitability models within the landscape to estimate potential population recovery targets (S4 Table). Given that we had no baseline densities and insufficient information on the covariates, which influence variations in the density of tigers and/or prey specific to the recovery, we did not perform PVA and hence were unable to assess the potential timelines of recovery. In S4 Table we provide the data sources and specify the methods used to derive the recovery targets specific to each site.

## Results and discussion

### Immense recovery potential, but incomplete baselines and uncertain timelines

**Fig 1:**
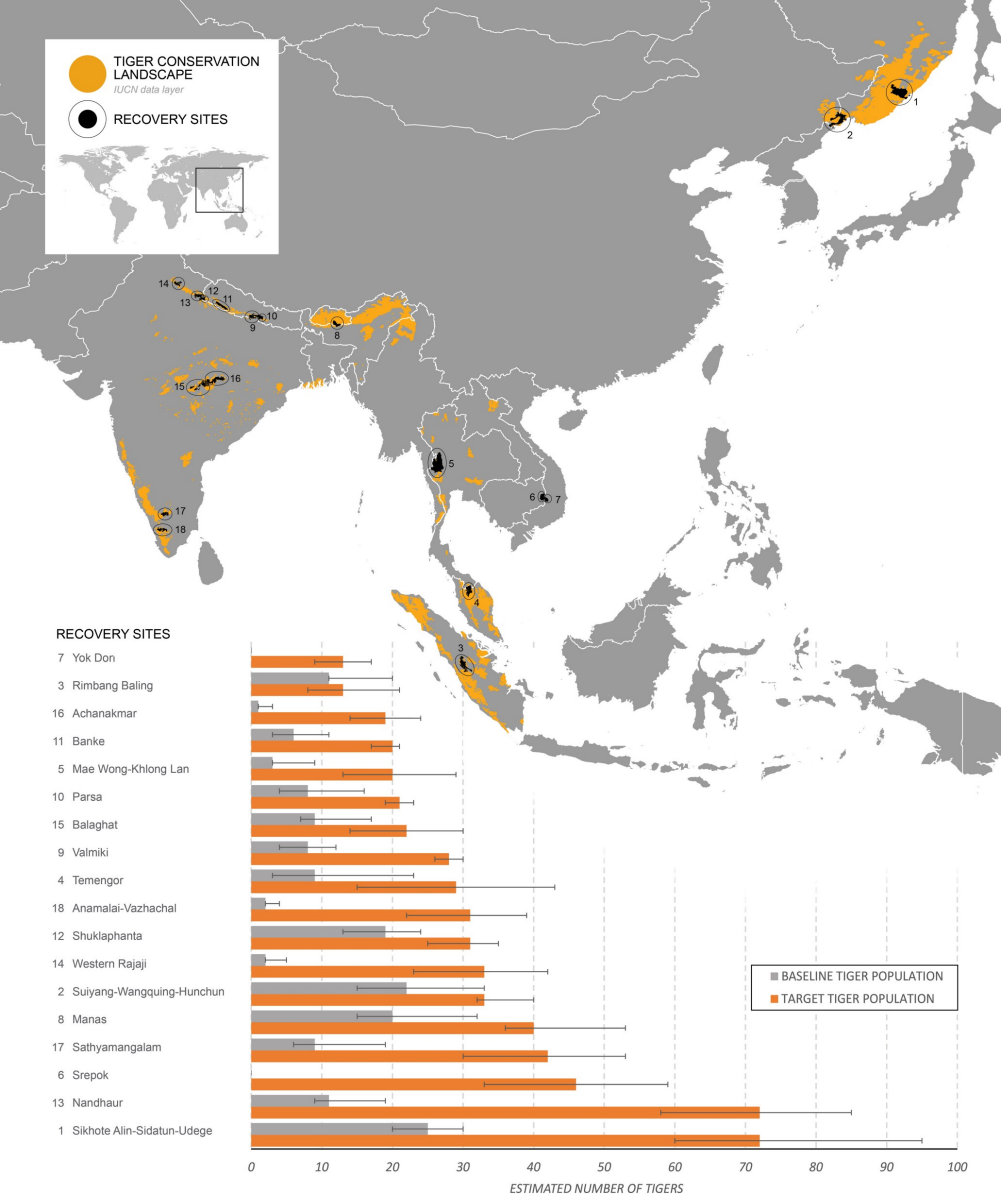
The 18 recovery sites across the tigers range. The location of the 18 recovery sites that have been delineated across 10 TRCs overlaid on the species range map [3,67]. Also depicted are the estimates of current and potential tiger population size for each site. Refer to S1 Fig to see the component parts of each of the 18 recovery systems.

Our assessment revealed that these 18 recovery sites (spread across 15 TCLs of differing priorities; S1 Table), which currently support 165 (118–277) tigers, could potentially harbour 585 (454–739) individuals (Fig 1, S5 Table), and thereby contribute towards a ~15% increase in the global population. This organizational stock-taking of project sites represents an important step towards fostering greater transparency and accountability for tiger conservation efforts, specifically aimed at achieving the global goal of doubling wild tiger numbers. Although we adopt multiple strategies to plan population recoveries under varying resolutions of data availability, the paucity of robust information on key demographic parameters of tigers and their prey and the factors affecting them limits our ability to assess potential timelines for recovery. Nevertheless, from a sub-set of sites (where robust site-level data were available, n = 4), it appears that recovery targets can be achieved in 15–20 years when natural prey is adequate, but may require considerably more time (30–50 years) in 15 of 18 sites where tiger recovery is contingent on the recovery of depleted prey populations.

A critical first step in planning population recovery is establishing relevant baselines. Estimates of tiger density were available from all 18 sites (Fig 1, S3 Table). In contrast, prey density estimates were available from just 7 sites. A reason for this is the prohibitively large survey effort required to estimate density in areas of low detection (e.g. sites in tropical evergreen and temperate forest habitats). Although alternate measures, such as proportion of area occupied by a species [68], cannot be used to estimate prey species abundance or set recovery targets, they can still be used to assess site-specific changes in species occurrence over time.

Given the challenges arising from paucity of site-level demographic parameters (in 14/18 sites), we set recovery targets based on the finest resolution at which data were available (Fig 1, S5 Table). Prior studies setting recovery targets (e.g. [16]) have assigned potential tiger densities to biomes/major habitat types at macroecological scales. These studies, however, did not account for heterogeneity in habitat features and management regimes within landscapes, even though such factors are known to drastically influence prey occurrence and abundance, and hence, recovery potential (e.g. [53]). In our assessment we employed multiple methods (S4 Table) using the best available data obtained from similar habitat types, adjacent source sites and other protected areas within the landscape to estimate potential population recovery targets, accounting for underlying spatial heterogeneity. Despite the shortcomings of our assessment, these represent conservative targets that could be established so that necessary conservation actions required to recover populations can be rapidly initiated. Nevertheless, we also suggest that simultaneously resources be made available for rigorous scientific assessments to generate these baselines and monitor trends and refine recovery targets across key tiger landscapes and all potential recovery sites.

Owing to the aforementioned data gaps, we were unable to gain a comprehensive understanding of site-specific factors influencing tiger and prey densities, and subsequently assess potential timelines for recovery. At two of the four sites with robust site-level ecological data (Shuklaphanta and western Rajaji), tiger densities were unnaturally depressed and current prey densities could support higher tiger numbers. However, these recoveries may occur over varying time periods owing to site-specific factors (S2 Fig). For instance, recovery may take 14 (95%CI 7–18) years in Shuklaphanta where the current density (6.3±0.18 individuals/100km^2^) is closer to the estimated carrying capacity (10 individuals/100km^2^), provided current rates of recovery prevail. The same may occur over 20 (95%CI 11–30) years in western Rajaji, where recovery is contingent upon a supplementation program and the restoration of key corridors to re-establish connectivity between the source and recovery sites. In Srepok, the population is estimated to require over 15 (95%CI 7–25) years to recover to a target of 46 upon reintroduction of tigers. However, suitable ecological and management conditions need to be attained at the site prior to reintroducing tigers [69]. Additionally, with small founding populations it is likely that inbreeding depression could affect the long-term viability of the population, thus, necessitating frequent supplementations to ensure population persistence. Finally, at the fourth site (Nandhaur), where tiger recovery is contingent on the recovery of prey (currently depressed by hunting), recovery is expected to take over 50 (95% CI 35–66) years. Accounting for uncertainty in parameter estimates indicate that these timelines could be greatly extended if adult survivorship is low (S2 Text). Even with adequate ecological knowledge, there could be critical delays in recovery timelines on account of weak institutional support, lacunae in conservation planning and ineffective leadership in a complex political arena (e.g.[70]).

Given that these 18 recovery sites span most major biomes that tigers inhabit across its range, our assessment has the potential to inform recovery planning at other sites at the global scale. Towards achieving this goal, we highlight the need to identify recovery sites outside known sources (while recognizing that sources must be secured [15]), to not just arrest declines but increase tiger numbers and thereby ensure that populations persist. We also recognize that in various landscapes, the demography of tigers may be governed by metapopulation dynamics driven by dispersal, colonization and gene flow. By delineating recovery systems, typically comprising a source site, recovery site and support region, we strongly advocate that that critical elements of the landscape are synergistically managed to meet the goals of recovery.

While we acknowledge that our approach is limited by the quality and resolution of available data, we believe it has several merits. First, our assessment can serve as a template to guide recovery planning in other sites and strategies of other conservation agencies. Second, we identified critical knowledge gaps that need to be addressed to plan, initiate and sustain population recoveries at these 18 sites and beyond. Finally, we anticipate that this assessment will be refined in due course as more robust information about key ecological and management drivers is made available on site-level population estimates for tigers and prey.

### Working towards the target

The practices which have proven to be effective in initiating and sustaining recoveries are well known and are either being implemented or proposed at the 18 sites ([8–11]; S6 Table). With both poaching and prey depletion shown to severely impact tiger populations [58,59], reduction of human-caused mortality is a ubiquitous high priority conservation action. Towards this end, it is essential that sites at least achieve prescribed protection and management standards (e.g. SMART and CA|TS; [71,72] see S6 Table for details). Ensuring effective protection and management of sites is often reliant on a multi-pronged approach including garnering community support to reduce critical threats [10], setting-up intelligence networks [73] and most importantly, establishing adaptive management systems [74,75]. Even at sites where the reintroduction/supplementation of tigers is required (e.g. Srepok, Yok Don and Western Rajaji; S6 Table), the success of the recovery program is contingent upon first improving protection and management standards [69,70].

Securing tiger meta-populations for long-term conservation requires maintaining functional connectivity [76]. This is hugely challenging both in terms of policy and implementation. Across these 18 recovery systems, key wildlife corridors have been degraded by land use change due to urbanization and infrastructure projects (S7 Table). Towards realizing the full recovery potential of the sites it is critical to align development objectives with conservation goals by conducting policy relevant assessments, pre-emptive advocacy for re-aligning infrastructure networks outside critical corridors, while also lobbying for upgraded law enforcement in these sites (e.g. [77]). Additionally, these recovery systems are embedded in some of the most densely populated regions of the world, with local populations showing high dependence on natural resources. Given the potential for increased conflict in areas with recovering tiger populations, it is critical to effectively manage deleterious impacts on human communities through suitable compensation programs and effective community-conservation initiatives to build enduring partnerships with communities for conservation [78].

The recovery of tiger populations is financially viable. While the costs of implementing site-based protection and monitoring measures have been previously estimated (e.g. [15]), differential returns on conservation investments in different countries owing to various factors (e.g. cost of labour or land; [79]) make such generalized estimations problematic. However, we suggest matching funds to the nearest source site within the TCL as an indicator of the funding gap that needs to be bridged, as this also accounts for the ecological, social and political contexts within which a site is embedded (S1 Table). Such funds can be sourced within the country (for example in India where the government has pledged 244.6 million USD for tiger conservation; [80]), or may have to be raised through international donors [80,81].

### Advocating an unwavering commitment towards tiger recovery

In conclusion, substantial population increase can be achieved across these recovery sites. However, it is evident that owing to the lack of robust baselines at the onset we, the signatories of the GTRP, may have committed to an ambitious goal for 2022. It is critical, as we cross this halfway mark, to commit our resources to achieving the biologically realistic targets for these sites even if the timelines are extended. At this juncture, population recoveries are contingent upon sustained political will and buy-in, responsive governance, adequate financial commitment and public support to ensure that the recommended actions are implemented in a timely manner. Finally, intensive biological monitoring based on consistent survey and analytical methods is essential to track changes and assess the effectiveness of the conservation efforts towards achieving the recovery targets [82].

## Supporting information

S1 Fig The 18 recovery systems. Spatial configuration of components of the 18 recovery systems within the larger tiger conservation landscapes across 10 TRCs [3,67], overlaid on a true-colour earth spatial layer [83].
(TIF)

S2 Fig Population viability analysis results. Results of the Population Viability Analysis for recovery at (A) Srepok, (B) Shuklaphanta, (C) Nandhaur and (D) Western Rajaji.
(TIF)

S3 Fig Connectivity analysis results. Connectivity analysis used in the delineation of the support regions for two recovery systems (A) Balaghat and (B) Achanakmar overlaid on a reclassified 2014 Landsat cloud-free image composite [84].
(TIF)

S1 Table Comparing recovery sites to sources. Comparison of management attention in terms of financial commitment and/or protection capacity between the recovery site and adjoining source or high density populations in the landscape.
(XLSX)

S2 Table Five recovery site classes. The five broad groups into which identified recovery sites fell based on known patterns of tiger and/or prey occurrence.
(XLSX)

S3 Table Tiger and prey baselines. Availability of baseline data and data sources of tigers and prey at the 18 recovery sites.
(XLSX)

S4 Table Setting recovery targets at sites. The two broad strategies used to estimate the potential population recovery targets at 18 recovery sites based on the resolution at which data on tiger and prey abundances, and associated habitat and threat covariates were available.
(XLSX)

S5 Table Baseline and target population sizes. Baseline and target tiger population densities and sizes for the 18 recovery sites.
(XLSX)

S6 Table Conservation actions. Conservation actions that are, either in place or proposed, to initiate tiger and prey recoveries at each of the sites.
(XLSX)

S7 Table Threats to connectivity. Threats to connectivity between the source and recovery site or in the support region.
(XLSX)

S8 Table Tiger demographic parameters. Summary of demographic parameters used in the Population Viability Analysis. Parameters derived from [1–4].
(XLSX)

S9 Table Prey demographic parameters. Demographic parameters of the six ungulate species used in the prey recovery model.
(XLSX)

S1 Text Tigers alive initiative. A description of the Tigers Alive Initiative, WWF.
(DOCX)

S2 Text Population viability analysis description. Description of the VORTEX model, unique set of scenarios simulated in the PVA and results of the sensitivity analysis.
(DOCX)
