# Supplementary figures and images for: Recovery planning towards doubling wild tiger Panthera tigris numbers: Detailing 18 recovery sites from across the range
Source: PLoS One. 2018 Nov 8;13(11):e0207114. doi: 10.1371/journal.pone.0207114 (PMC6224104; doi:10.1371/journal.pone.0207114)

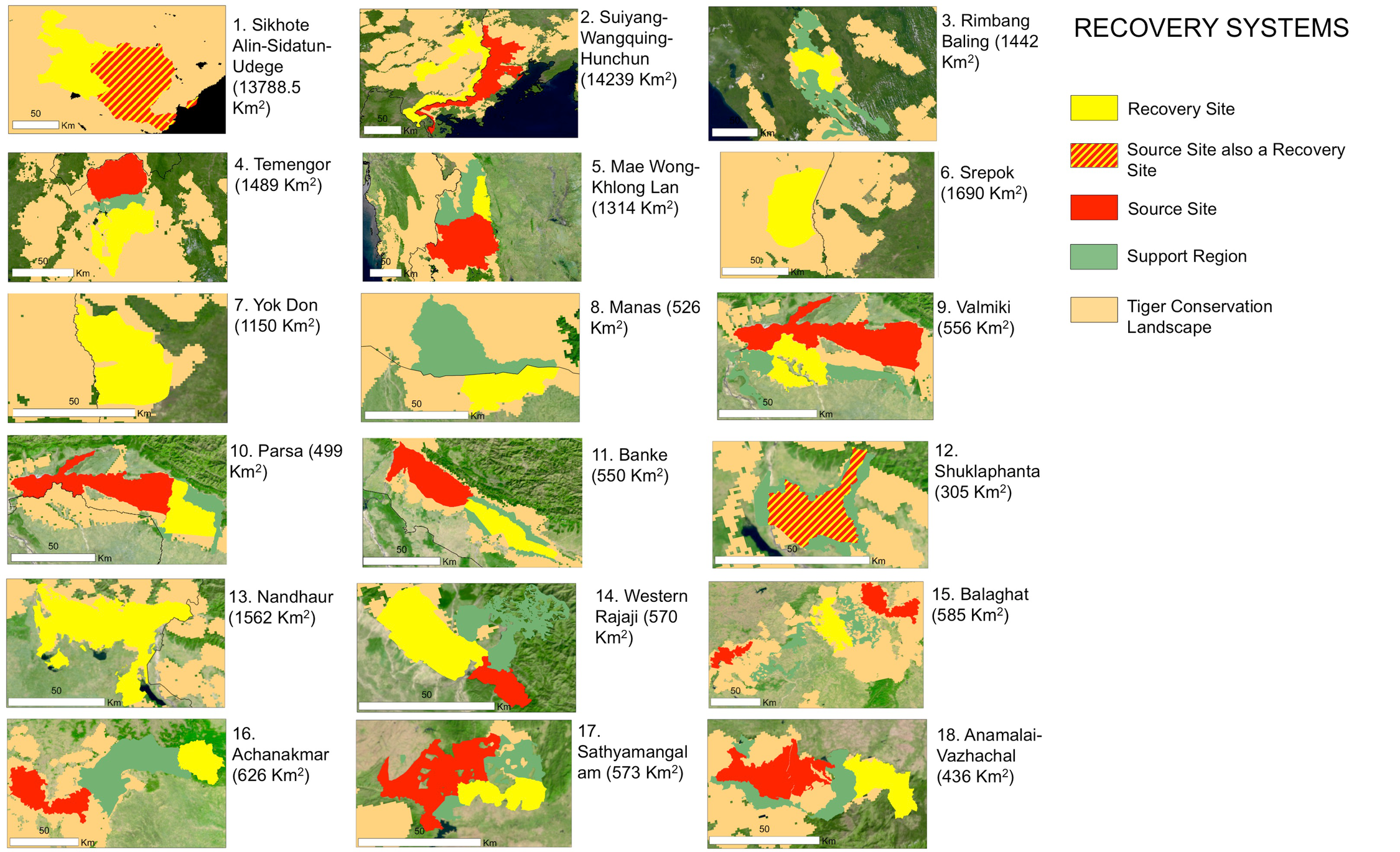

Supplement: S1 Fig — Spatial configuration of components of the 18 recovery systems within the larger tiger conservation landscapes across 10 TRCs [3,67], overlaid on a true-colour earth spatial layer [83]. (TIF) [file pone.0207114.s001.tif]

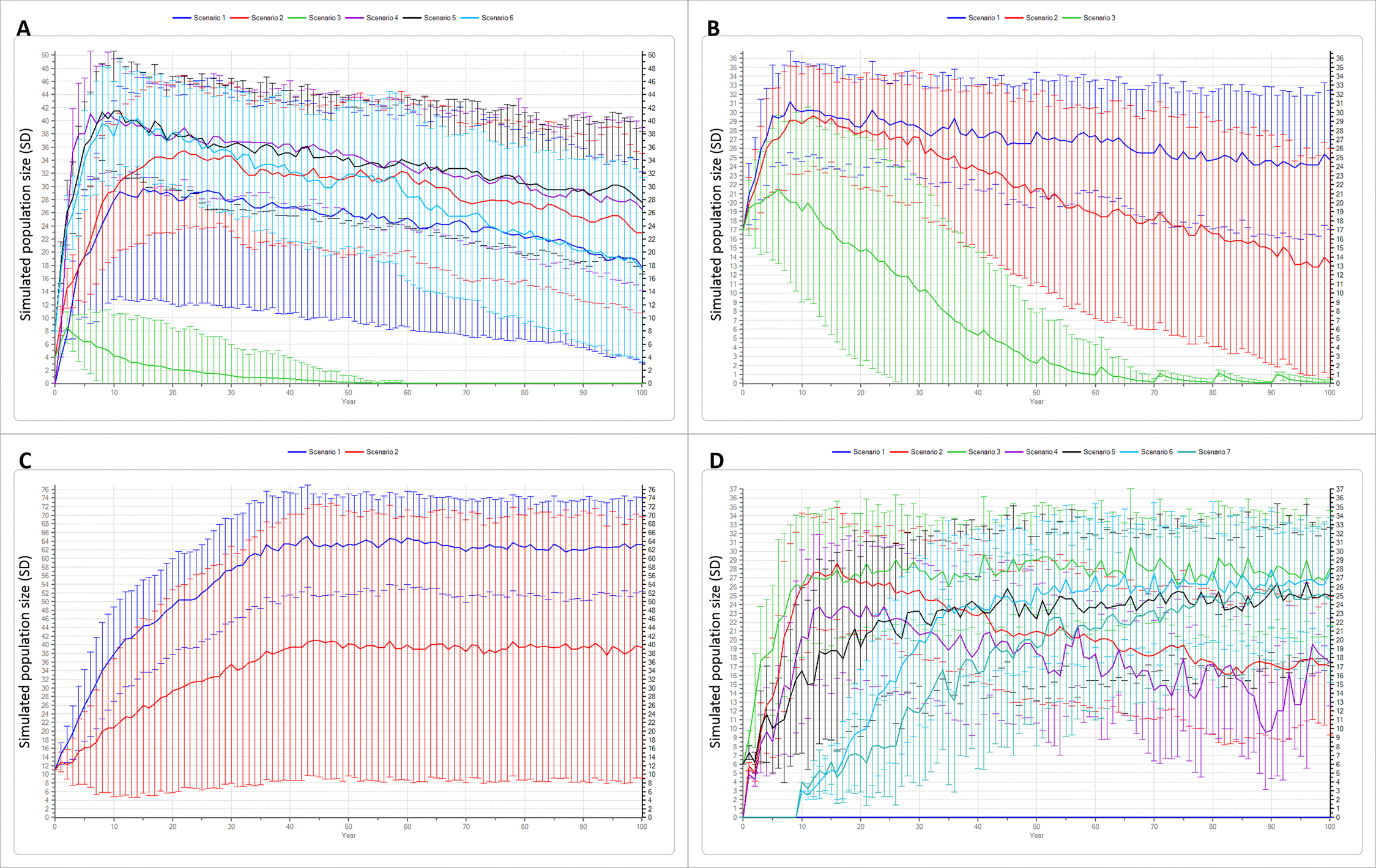

Supplement: S2 Fig — Results of the Population Viability Analysis for recovery at (A) Srepok, (B) Shuklaphanta, (C) Nandhaur and (D) Western Rajaji. (TIF) [file pone.0207114.s002.tif]

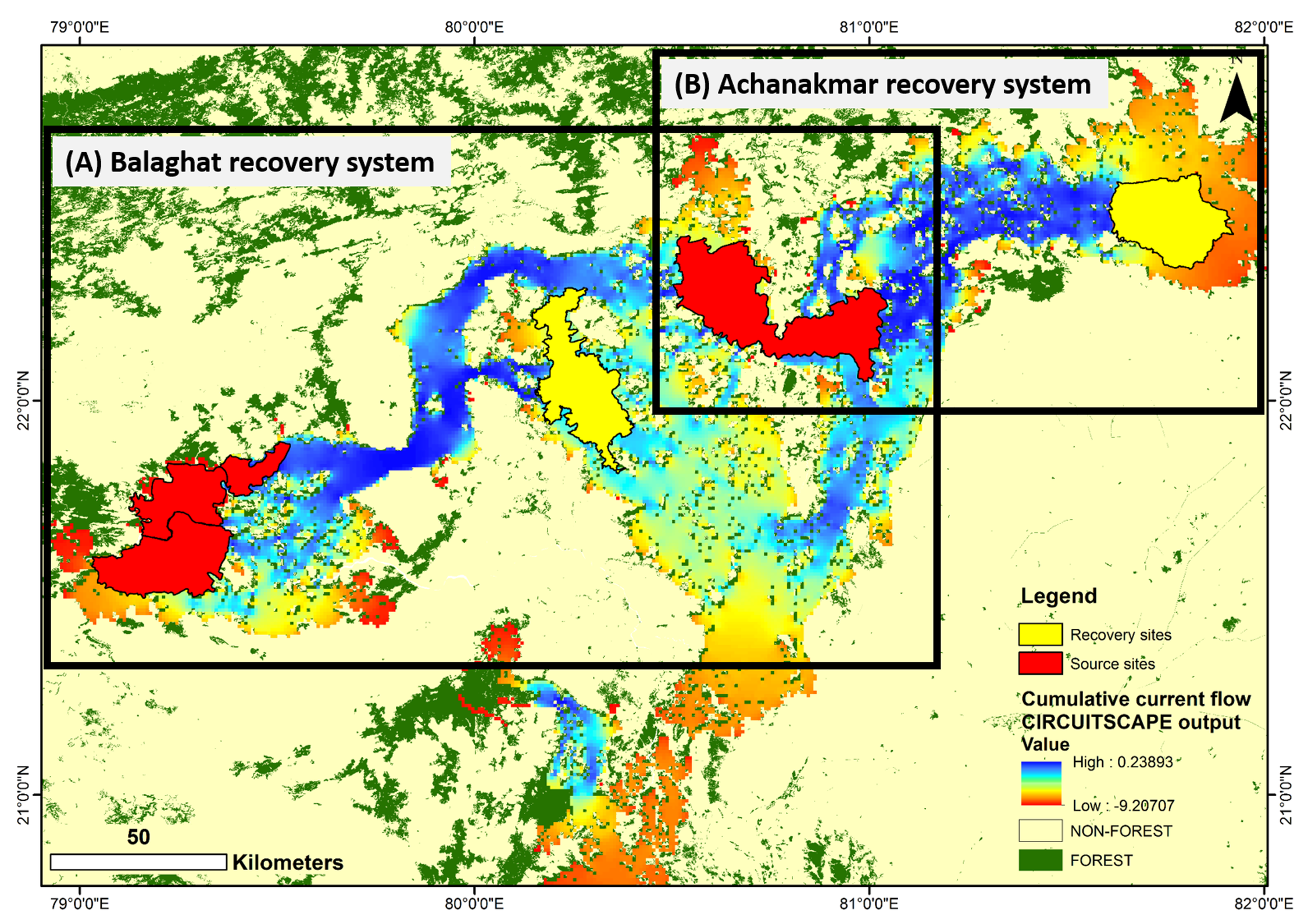

Supplement: S3 Fig — Connectivity analysis used in the delineation of the support regions for two recovery systems (A) Balaghat and (B) Achanakmar overlaid on a reclassified 2014 Landsat cloud-free image composite [84]. (TIF) [file pone.0207114.s003.tif]
