# Supplementary material for: Recovery planning towards doubling wild tiger Panthera tigris numbers: Detailing 18 recovery sites from across the range
Source: PLoS One. 2018 Nov 8;13(11):e0207114. doi: 10.1371/journal.pone.0207114 (PMC6224104; doi:10.1371/journal.pone.0207114)
Supplement: S1 Text — A description of the Tigers Alive Initiative, WWF. (DOCX) [file pone.0207114.s013.docx]

# **S1 Text. Tigers Alive Initiative.**

## **A description of the Tigers Alive Initiative, WWF**

The Tigers Alive Initiative is a programme of work aimed to recover the global wild tiger population working at multiple scales from the field level to the global policy arena. The programme primarily focuses on ensuring that sites supporting or potentially supporting tiger populations are effectively managed to sustain or recover tiger populations using the CA|TS standards to measure the effectiveness of the management. It provides financial and technical support and capacity in cooperation with local partners, often government agencies, to improve monitoring of tigers and their prey and protection from poaching, to halt habitat loss and degradation, to reduce the negative impacts of human-tiger conflict and build support by communities for tiger conservation. Using these sites as the building blocks, the Initiative aims to maintain larger intact tiger landscapes by supporting permeable and supportive land-uses and reduce/minimize the adverse impacts of infrastructure projects that have the potential to fragment or degrade tiger habitats. Outside of tiger landscapes, the initiative further aims to achieve tiger conservation by raising political and financial support through the national and international network of WWF and by working on the drivers that lead to habitat loss and demand for tigers and their parts. The Initiative was established in 2009, encompasses all of the WWF network investment in tiger conservation, operates in 13 tiger landscapes and focuses effort in approximately 75 protected areas in 12 countries.

See <http://tigers.panda.org> for further details.
