# Supplementary material for: Recovery planning towards doubling wild tiger Panthera tigris numbers: Detailing 18 recovery sites from across the range
Source: PLoS One. 2018 Nov 8;13(11):e0207114. doi: 10.1371/journal.pone.0207114 (PMC6224104; doi:10.1371/journal.pone.0207114)
Supplement: S2 Text — Description of the VORTEX model, unique set of scenarios simulated in the PVA and results of the sensitivity analysis. (DOCX) [file pone.0207114.s014.docx]

# **S2 Text. Population Viability Analysis description.**

## **Description of the VORTEX model, unique set of scenarios simulated in the PVA and results of the sensitivity analysis.**

### **The VORTEX model**

VORTEX is an individual-based simulation model that incorporates both deterministic forces as well as environmental, demographic and genetic stochasticity on the viability of species populations [1–3]. Individual behaviour (i.e. surviving or reproducing) is drawn from a binomial distribution with the mean set by the inputted life history data and the variation in the carrying capacity (*K*) is modelled as a normal distribution [2,3]. Primarily developed to simulate extinction vortices that threaten the persistence of small vertebrate populations of low fecundity and long life spans, VORTEX is highly suitable for modelling tiger populations. However, studies caution the use of such Population Viability Analysis (PVA) model in conservation planning but do recognize that such analyses help explore the implications of model assumptions on extinction probabilities [4,5]. We have used VORTEX in such an exploratory capacity. Our primary aim of using the model is to evaluate the qualitative differences in tiger recovery scenarios to help us broadly characterize the conditions required to implement a successful conservation program. Additionally, we infer on the potential timelines for species recoveries across sites and scenarios as an important outcome, rather than the quantitative detail.

We used PVAs to evaluate population growth and assess the probability of survival by tigers to the proposed recovery efforts at four sites. Across all sites and scenarios, we used life history data on tigers that has been previously used in similar analysis [6–11]. We started all our models at the initial population size (*N_0_*) specific to each of the sites and set site-specific carrying capacities (*K*) (See “**Description of the unique set of scenarios simulated in the PVA”** for further details). Although the role of inbreeding depression in the extinction of wild populations is debatable, it is often considered important in conservation planning due to problems caused by recessive lethal alleles. However, in the absence of appropriate data we used default values in VORTEX for inbreeding [12]. We assumed half the number of adult males and females contributed to the breeding pool (with SD 10). Stage specific survival estimates were derived from literature [6–8,10,11,13,14] (S8 Table). These estimates have been derived from a range of studies. While it is expected that individual populations may behave differently, we have been forced to use this data rather than statistics from a single tiger population. While density dependent reproduction for populations below *K* is expected to increase survival of populations (e.g. [15]), the lack of suitable data prevented us from incorporating density dependence. As mentioned previously, our primary aim was to broadly characterize the conditions required to recover and sustain tiger population at these four sites (where we had the data to do so). Hence, we did not model periodic environmental catastrophes, although such events may be important. An example of such an event affecting large carnivores is the Canine Distemper Virus epidemic that almost wiped out the Serengeti lion population in 1994 [16]. With the exception of density dependence, all our other assumptions are conservative, tending to increase the survival of populations. For the analysis, all simulations were run for 500 iterations for a period of 100 years and extinction was defined as the condition when the population is reduced to only one sex. A unique set of scenarios was evaluated for each of the sites based on relevant prior information. It is to be noted that these are not an exhaustive evaluation of all feasible management options, but a suite of possible broad alternatives to help guide future conservation action.

### **Description of the unique set of scenarios simulated in the PVA**

#### **Srepok Wildlife Sanctuary**

Srepok Wildlife Sanctuary (SWS) is situated in a connected protected area complex (>14,000km²) known as in the Eastern Plains Landscape. SWS (3724km²) was identified as one of the priority tiger recovery sites, with a focus on the core areas (1,700km²). However, the site or the surrounding protected areas the landscape currently does not support a breeding population of tigers. The last confirmed sighting within the landscape and the country was a photographic record of a tigers at the site in SWS comes from in 2007 [17], while a survey in 2010 confirmed the presence of at least 1 tiger from tracks. However, since then there have been no records of confirmation despite systematic effort with camera traps and scat detection dogs. It is highly likely that that wild tigers are extinct in Cambodia. Therefore, a reintroduction program has been suggested to repopulate this site and the surrounding landscape with tigers [18]. WWF commissioned a study to examine the feasibility of a restoration programme in the landscape with the objectives of reintroduction if tigers were entirely extirpated or reinforcement if a few tigers still inhabited the area [19]. This assessment developed a population viability analysis to assess various reintroduction/supplementation scenarios, and we used the same parameters to replicate the scenarios here. The initial population size (*N_0_*) was set at zero and the carrying capacity (*K*) at 46 (SD 12). Simulations were carried out to evaluate 6 scenarios: Scenario 1 - A founding population of four individuals (3 females and 1 male); Scenario 2 - A founding population of four individuals (3 females and 1 male) and supplementation of two males within first year; Scenario 3 - A founding population of four individuals (3 females and 1 male), supplementation of two males within first year and poaching of one animal every three years; Scenario 4 - A founding population of eight individuals (6 females and 2 males); Scenario 5 - A founding population of eight individuals (6 females and 2 males) and supplementation of one pair every three years for three consecutive terms; Scenario 6 - A founding population of eight individuals (6 females and 2 males), supplementation of one pair every three years for three consecutive terms and poaching of one individual every three years.

#### **Shuklaphanta**

The tiger population in the Shuklaphanta Wildlife Reserve, Nepal has been monitored since 1999 using camera trap surveys and capture-recapture analyses [20]. From 1999 to 2001, *adhoc* camera trapping in the reserve resulted in the photo-capture of approximately 23 individual adult tigers. In subsequent surveys (2001-02 and 2002-03), 17 and 18 individuals were captured, respectively. However, camera trapping surveys from 2004-05 to 2007-08 revealed a considerable decrease in abundance by 30% and 62%, respectively. Estimates of the relative abundance of key tiger prey species indicate that populations were comparatively stable over the same period. Tiger Task Team [21] concluded that the decline was primarily due to poaching from this wildlife reserve. However, with the re-establishment of government rule in Nepal and the reinstatement of a stringent protection regime (since 2010), the tiger population appears to have grown; 17 tigers were photo-captured in 2012-13, and the species' density was estimated to be 6.3±0.18 individuals/100km^2^. Based on this the initial population size (*N_0_*) was set at 17 and the carrying capacity (*K*) at 34 (SD 4) (see S4 Table for details). Simulations were carried out to evaluate 3 scenarios: scenario 1 – only demographic parameters; scenario 2 – only demographic parameters and poaching of two individual every three years; scenario 3 **-** only demographic parameters and poaching of two individuals every year (estimated rate of poaching at Shuklaphanta;[21]).

#### **Nandhaur**

The Nandhaur section of the Terai Arc Landscape covering 1562 km^2^, is bound by river Gola to the west, Sharda to the east and river Ladhya to the north, while the southern boundary is fringed by agricultural fields and urban settlements. The newly designated Nandhaur WLS covering 274 km^2^ is surrounded by Champawat, Haldwani and Terai East reserve (multiple-use) forests. Disturbance was reported to be high, primarily by way of prey poaching, livestock grazing and non-timber forest produce collection emanating from villages along both the northern and southern boundaries of the PA, and the pastoralist Gujjar community which resides and herds buffaloes within these forests. The critical threat limiting tiger recovery in the site is prey poaching [22]. Mann *et al.*[22] estimated densities of ungulate prey species (barking deer, chital, wild pig, sambar, nilagai, goral and serow) to be 7.08 (1.44)/km^2^ and density and abundance for tigers to be 0.71adult tigers/100km^2^ or 11 individuals respectively. These are drastically lower compared to sites with comparable habitats within the same landscape [23,24], likely because of poaching and human pressure on tiger and prey habitats [22,25]. Therefore, strengthening enforcement to curb illegal hunting was thought to be critical for tiger and prey population recovery in Nandhaur.

To simulate population dynamics in response to conservation measures, we adopted a two-step approach. First we simulated recovery in prey populations in response to curbing wild prey hunting through stringent enforcement of the law. Then we assessed timelines of recovery of tiger populations in response to recovering prey population. To simulate prey recovery trajectories we constructed species-specific population viability models. These models were constructed incorporating the effects of inbreeding depression by using the default values in VORTEX for inbreeding [12]. Species specific population parameters were obtained from a wide range of studies and sources (see S9 Table for details). Initial population sizes (*N_0_*) were set as the species-specific densities derived by Mann *et al.*[22] and a stable age distribution and species-specific potential carrying capacities (*K*) were set by extending the predictions of a spatially-explicit prey population density model developed by Harihar *et al.*[23]. The predictive General Additive Model developed using Density Surface Modeling for wild ungulate prey in the western Terai Arc Landscpe [23], which modelled for the effects of habitat (elevation, ruggedness, vegetation productivity and vegetation seasonality) and anthropogenic covariates (distance to forest settlements and distance to forest edge) on prey densities, was extended onto Nandhaur. The model predicted significantly higher densities for the wild ungulates, further confirming that heightened prey poaching is the more probable cause for the existing low prey densities. We built the species-specific PVA models under the assumption that stringent protection would facilitate for the recovery of tiger prey species. These PVA results were then used to estimate potential carrying capacity for tigers following the scaling equation of Karanth *et al.* [26]. This rate of change in carrying capacity was then used to simulate recovery scenarios for tigers. The recovery of tigers was evaluated under 2 scenarios. Scenario 1 **–** Recovery of tigers with a prey recovery rate corresponding to a 2.1% annual increase in carrying capacity over 35 years. Scenario 2 **–** Recovery of tigers with a prey recovery rate corresponding to a 2.1% annual increase in carrying capacity over 35 years, with effects of tiger poaching of two individual every three years.

#### **Western Rajaji**

In the western extreme of the Terai Arc Landscape (the north-western range limit of tigers), tigers occupy 58% (of 4,109km^2^) of available habitat. However, the landscape has been cleaved into two near-disjunct units termed Tiger Habitat Blocks (THBs I and II;[25]), isolated from one another on account of the expansion of Haridwar town and a busy highway and railway line [27]. While tigers occupy 88.5% of THB II (encompassing the protected areas of eastern Rajaji and Corbett Tiger Reserves), tigers in THB I (encompassing western Rajaji Tiger Reserve) occupy a mere 17%[28]. Following the reduction of human pressure from key wildlife habitats, eastern Rajaji Tiger Reserve has witnessed a significant recovery in the population of tiger. Studies have primarily attributed this to (a) minimization of anthropogenic pressures which included the voluntary resettlement of *Gujjars* that led to disturbance free habitats safe for tiger to breed in, and (b) connectivity of eastern Rajaji Tiger Reserve with Corbett Tiger Reserve through the Rajaji-Corbett corridor (Lansdowne Forest Division) that led to the immigration and subsequent colonization of individuals[29,30]. Even though settlements from most parts of western Rajaji Tiger Reserve (400km^2^) have also been relocated, and prey populations are sufficiently large to support 33 adult tigers (SD 7) [23], the species' population has not recovered. Long-term (15 years; 1994-95 to 2008-09) sign survey data [27], reveals that the occurrence of tigers with the park has been declining. With photographic evidence suggesting the presence of just two females [28], and no signs of breeding having been recorded since 2006, supplementation is critical to ensure the persistence as well as augmentation of the population. Genetic studies [31,32] suggest that this sub-population is identical to other populations in the Terai Arc Landscape and therefore, release stock could be from regions adjoining a high density source such as Corbett Tiger Reserve.

In our PVA models for western Rajaji national Park, we (a) set the initial population size (*N_0_*) at 2 (both females), (b) set the carrying capacity (*K*) at 33 and (c) evaluated population trajectories under 7 scenarios. Scenario 1– Isolation, (carry out no management intervention to recover the population); Scenario 2 – Supplement the population with four individuals (3 females and 1 male) over a span of 10 years; Scenario 3 - Supplement the population with four individuals (3 females and 1 male) over a span of 10 years and restore connectivity across the Chilla-Mothichur corridor to allow for natural dispersal and colonization of three individuals every five years from eastern Rajaji Tiger Reserve; Scenario 4 - Supplement the population with four individuals (3 females and 1 male) over a span of 10 years, with effects of poaching (loss of 2 individuals every three years); Scenario 5 - Supplement the population with four individuals (3 females and 1 male) over a span of 10 years and restore connectivity across the Chilla-Mothichur corridor to allow for natural dispersal and colonization of three individuals every five years from eastern Rajaji Tiger Reserve with effects of poaching (loss of 2 individuals every three years); Scenario 6 – Restore connectivity across the Chilla-Mothichur corridor to allow for natural dispersal and colonization of three individuals every five years from eastern Rajaji Tiger Reserve (starting from the tenth year); Scenario 7 - Restore connectivity across the Chilla-Mothichur corridor to allow for natural dispersal and colonization of three individuals every five years from eastern Rajaji Tiger Reserve (starting from the tenth year) with effects of poaching (loss of 2 individuals every three years).

### **Results of the sensitivity analysis**

Sensitivity analysis revealed a high degree of uncertainty in the projections owing to variability in the parameters. In *Srepok Wildlife Sanctuary*, Scenario 2 (A founding population of four individuals (3 females and 1 male) and supplementation of two males within first year), Scenario 4 (A founding population of eight individuals (6 females and 2 male)) and Scenario 5 (A founding population of eight individuals (6 females and 2 male) and supplementation of one pair every three years for three consecutive terms) ranked similarly when accounting for parameter uncertainty. Under low survivorship estimates, the timelines for recovery were extended to about 42 (95% CI 38-45) years as opposed to 15 (95%CI 7-25) years. In *Shuklaphanta,* while the results were sensitive to uncertainty. Scenario 1 (characterising current recovery trajectory) was always ranked the best. Under low survivorship estimates, the timelines for recovery were extended to about 24 years (95% CI 20-29) as opposed to 14 (95%CI 7-18) years. In *Nandhaur,* the results were sensitive to uncertainty yet the timelines for recovery were extended to about 78 (95% CI 45-111) years as opposed to 50 (95% CI 35-66) years. Finally in *Western Rajaji,* Scenario 3 (supplement the population with four individuals, .i.e.3 females and 1 male, over a span of 10 years and restore connectivity across the Chilla-Mothichur corridor to allow for natural dispersal and colonization of individuals from eastern Rajaji Tiger Reserve) and Scenario 6 (restore connectivity across the Chilla-Mothichur corridor to allow for natural dispersal and colonization of individuals from eastern Rajaji Tiger Reserve over a 10 year period) ranked similarly when accounting for parameter uncertainty. Under low survivorship estimates, the timelines for recovery were extended to about 52 (95% CI 34-66) years as opposed to 20 (95%CI 11-30).

# **References**

1. Lacy RC, Pollak JP. Vortex: A stochastic simulation of the extinction process. Version 10.0. vortex10. org/Vortex10. aspx [15 May 2014]. Brookfield, Illinois, USA: Chicago Zoological Society,; 2014.

2. Lacy RC. VORTEX: a computer simulation model for population viability analysis. Wildl Res. CSIRO; 1993;20: 45–65.

3. Lacy RC. Structure of the VORTEX simulation model for population viability analysis. Ecol Bull. JSTOR; 2000; 191–203.

4. Coulson T, Mace GM, Hudson E, Possingham H. The use and abuse of population viability analysis. Trends Ecol Evol. Elsevier; 2001;16: 219–221.

5. Lindenmayer DB, Possingham HP, Lacy RC, McCarthy MA, Pope ML. How accurate are population models? Lessons from landscape‐scale tests in a fragmented system. Ecol Lett. Wiley Online Library; 2003;6: 41–47.

6. Horev A, Yosef R, Tryjanowski P, Ovadia O. Consequences of variation in male harem size to population persistence: Modeling poaching and extinction risk of Bengal tigers (Panthera tigris). Biol Conserv. 2012;147: 22–31.

7. Karanth KU, Stith BM. Prey depletion as a critical determinant of tiger population viability. In: Jackson P, Seidenstricker J, Christie S, editors. Riding the Tiger: Tiger conservation in human dominated landscapes. Cambridge University Press; 1999. pp. 100–113.

8. Kenney JS, Smith JLD, Starfield AM, McDougal CW. The long term effects of tiger poaching on population viability. Conserv Biol. 1995;9: 1127–1133.

9. Carter N, Levin S, Barlow A, Grimm V. Modeling tiger population and territory dynamics using an agent-based approach. Ecol Modell. 2015;312: 347–362. doi:https://doi.org/10.1016/j.ecolmodel.2015.06.008

10. Kenney J, Allendorf FW, McDougal C, Smith JLD. How much gene flow is needed to avoid inbreeding depression in wild tiger populations? Proc R Soc B. The Royal Society; 2014;281: 20133337.

11. Smith JLD, McDougal C. The contribution of variance in lifetime reproduction to effective population size in tigers. Conserv Biol. 1991;5: 484–490.

12. O’Grady JJ, Brook BW, Reed DH, Ballou JD, Tonkyn DW, Frankham R. Realistic levels of inbreeding depression strongly affect extinction risk in wild populations. Biol Conserv. Elsevier; 2006;133: 42–51.

13. Chapron G, Miquelle DG, Lambert A, Goodrich JM, Legendre S, Clobert J. The impact on tigers of poaching versus prey depletion. J Appl Ecol. 2008;45: 1667–1674.

14. Jones KE, Bielby J, Cardillo M, Fritz SA, O’Dell J, Orme CDL, et al. PanTHERIA: a species-level database of life history, ecology, and geography of extant and recently extinct mammals: Ecological Archives E090-184. Ecology. 2009;90: 2648.

15. Chapman AP, Brook BW, Clutton-Brock TH, Grenfell BT, Frankham R. Population viability analyses on a cycling population: a cautionary tale. Biol Conserv. Elsevier; 2001;97: 61–69.

16. Roelke-Parker ME, Munson L, Packer C, Kock R, Cleaveland S, Carpenter M, et al. A canine distemper virus epidemic in Serengeti lions (Panthera leo). Nature. Nature Publishing Group; 1996;379: 441.

17. Gray TNE, Phan C, Pin C, Prum S. Establishing a monitoring baseline for threatened large ungulates in eastern Cambodia. Wildlife Biol. BioOne; 2012;18: 406–413.

18. Gray TNE, Crouthers R, Ramesh K, Vattakaven J, Borah J, Pasha MKS, et al. A framework for assessing readiness for tiger Panthera tigris reintroduction: a case study from eastern Cambodia. Biodivers Conserv. 2017;26: 2383–2399. doi:10.1007/s10531-017-1365-1

19. Launay F, Cox N, Baltzer M, Tepe T, Seidensticker J, Christie S, et al. Preliminary Study of the Feasibility of a Tiger Restoration Programme in Cambodia’s Eastern Plains. Phnom Penh; 2013.

20. Thapa K, Wikramanayake E, Malla S, Acharya KP, Lamichhane BR, Subedi N, et al. Tigers in the Terai: Strong evidence for meta-population dynamics contributing to tiger recovery and conservation in the Terai Arc Landscape. Gratwicke B, editor. PLoS One. 2017;12: e0177548. doi:10.1371/journal.pone.0177548

21. Mishra N, Karki JB, Pokhrel CP, Thapa K. Tiger monitoring in Suklaphanta Wildlife Reserve, submitted to Suklaphanta Wildlife Reserve. Kanchanpur, Nepal; 2008.

22. Mann R, Warrier R, Chanchani P. Status of Tiger, Leopard and Prey in Nandhaur Valley, Baseline estimates from the sub-Himalayan Nandhaur region of Uttarakhand, India. New Delhi; 2013.

23. Harihar A, Pandav B, MacMillan DC. Identifying realistic recovery targets and conservation actions for tigers in a human-dominated landscape using spatially explicit densities of wild prey and their determinants. Divers Distrib. 2014;20: 567–578.

24. Jhala Y V., Qureshi Q, Gopal R. The status of tigers in India 2014. New Delhi & Dehradun; 2015.

25. Johnsingh A, Ramesh K, Qureshi Q, David A, Goyal S, Rawat G, et al. Conservation status of tiger and associated species in the Terai Arc Landscape, India. Dehradun; 2004.

26. Karanth KU, Nichols JD, Kumar N, Link WA, Hines JE. Tigers and their prey: Predicting carnivore densities from prey abundance. Proc Natl Acad Sci. 2004;101: 4854–4858.

27. Harihar A, Ghosh-Harihar M, MacMillan DC. Losing time for the tiger Panthera tigris: delayed action puts a globally threatened species at risk of local extinction. Oryx. 2018;52: 78–88. doi:10.1017/S0030605317001156

28. Harihar A, Pandav B. Influence of connectivity, wild prey and disturbance on occupancy of tigers in the human-dominated western Terai Arc Landscape. Hayward M, editor. PLoS One. Public Library of Science; 2012;7: e40105. doi:10.1371/journal.pone.0040105

29. Harihar A, Pandav B, Goyal SP. Responses of tiger (Panthera tigris) and their prey to removal of anthropogenic influences in Rajaji National Park, India. Eur J Wildl Res. 2009;55: 97–105. doi:10.1007/s10344-008-0219-2

30. Harihar A, Pandav B, Goyal SP. Responses of leopard Panthera pardus to the recovery of a tiger Panthera tigris population. J Appl Ecol. 2011;48: 806–814. doi:10.1111/j.1365-2664.2011.01981.x

31. Sharma R, Stuckas H, Bhaskar R, Rajput S, Khan I, Goyal S, et al. mtDNA indicates profound population structure in Indian tiger (Panthera tigris tigris). Conserv Genet. Springer Netherlands; 2009;10: 909–914. doi:10.1007/s10592-008-9568-3

32. Mondol S, Karanth KU, Ramakrishnan U. Why the Indian Subcontinent Holds the Key to Global Tiger Recovery. PLoS Genet. Public Library of Science; 2009;5: e1000585. Available: http://dx.doi.org/10.1371%2Fjournal.pgen.1000585
